# Castleman disease of plasma cell type accompanied with bronchiolitis obliterans: a case report and review of the literature

**DOI:** 10.1186/s13256-023-04285-2

**Published:** 2023-12-12

**Authors:** Qingyuan Zhu, Shuiyou Wang

**Affiliations:** Department of Respiratory Diseases, Quzhou TCM Hospital at the Junction of Four Provinces Affiliated to Zhejiang Chinese Medical University, Quzhou, 324002 Zhejiang Province China

**Keywords:** Castleman disease, Bronchiolitis obliterans, Immunosuppressive medication, Case report, Follow-up

## Abstract

**Background:**

Castleman disease, also known as giant lymph node hyperplasia or angiofollicular lymph node hyperplasia, is a highly heterogeneous clinicopathological entity that belongs to the family lymphoproliferative disorders. Castleman disease accompanied by bronchiolitis obliterans is uncommon and often poses a great diagnostic challenge, which is easily confused with respiratory diseases and impeding the correct diagnosis and treatment. The main aim in presenting such rare case studies is to raise awareness and expand the diagnostic horizon of clinicians for appropriate management.

**Case presentation:**

Here, we present a 69-year-old Chinese male who was admitted to our hospital due to right chest pain for 6 months, accompanied by cough, expectoration, and fever. Laboratory examinations revealed elevated immunoglobulin G and C-reactive protein, and normal serum levels of tumor markers and interleukin-6. Computed tomography scan detected diffuse bronchial wall thickening and patchy area of air trapping consistent with small airway disease. Pulmonary function test showed mild small airway obstructive ventilation dysfunction and moderate decrease in diffusion capacity. The pathological result of the right axillary lymph node was consistent with the plasma cell type Castleman disease. According to the above examinations, the patient was finally diagnosed with the plasma cell type Castleman disease accompanied with bronchiolitis obliterans. He received immunosuppressive medication after surgery and has been followed up for 11 months. Now the patient is currently in stable condition without recurrence.

**Conclusion:**

Castleman disease is a rare lymphoproliferative disorder with a variety of symptoms. At present, the treatment of Castleman disease accompanied with bronchiolitis obliterans is mostly based on experiences or previous case reports, and there is no standard treatment. Here, we report an uncommon case of Castleman disease accompanied with bronchiolitis obliterans in which the patient received immunosuppressive medication after surgery and has been followed up for 11 months without experiencing a recurrence, which may deepen and extend our understanding of this disease.

## Introduction

Castleman disease (CD) is a rare lymphoproliferative disorder that was first described by Benjamin Castleman in 1956 [1, 2]. According to the area of lymph node involvement, CD can be divided into unicentric Castleman disease (UCD) and multicentric Castleman disease (MCD). Its histopathology can be divided into hyaline vascular type CD (HV-CD), plasma cell type CD (PC-CD), and mixed type CD [3]. There is no significant gender difference in the incidence of CD [4]. Despite increasing reports on CD, the condition remains difficult to diagnose, particularly when it is accompanied with bronchiolitis obliterans (BO). BO is a rare and ominous complication of Castleman disease. At present, there is no standard treatment for this disease and the mortality is high. To control the progression of BO, the effective adjuvant therapy should be given after resection of the tumor, such as glucocorticoid or immunosuppressive medication. We report a case of PC-CD accompanied by BO that presented with respiratory systemic manifestations and was initially diagnosed as a pulmonary infection.

## Case presentation

**Fig. 1:**
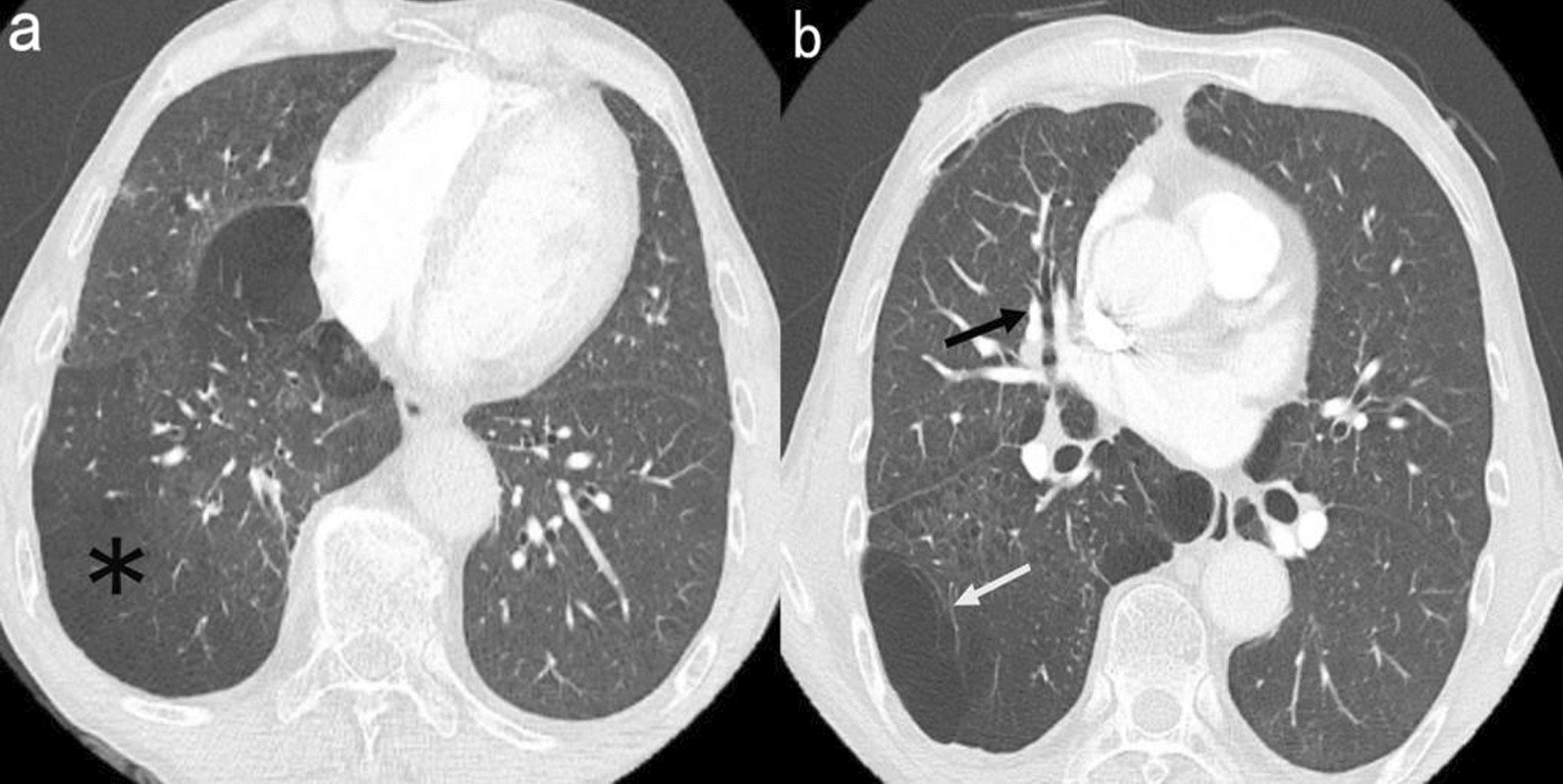
Axial images from chest computed tomography; **a** chest computed tomography scan presented patchy area of air trapping (_*_), **b** chest computed tomography scan revealed diffuse bronchial wall thickening with bronchiectasis (black arrow), local emphysema (white arrow), and leaf interval thickening; these findings are consistent with bronchiolitis obliterans, and the other typical sign of bronchiolitis obliterance, mosaic perfusion pattern, was not observed

**Fig. 2:**
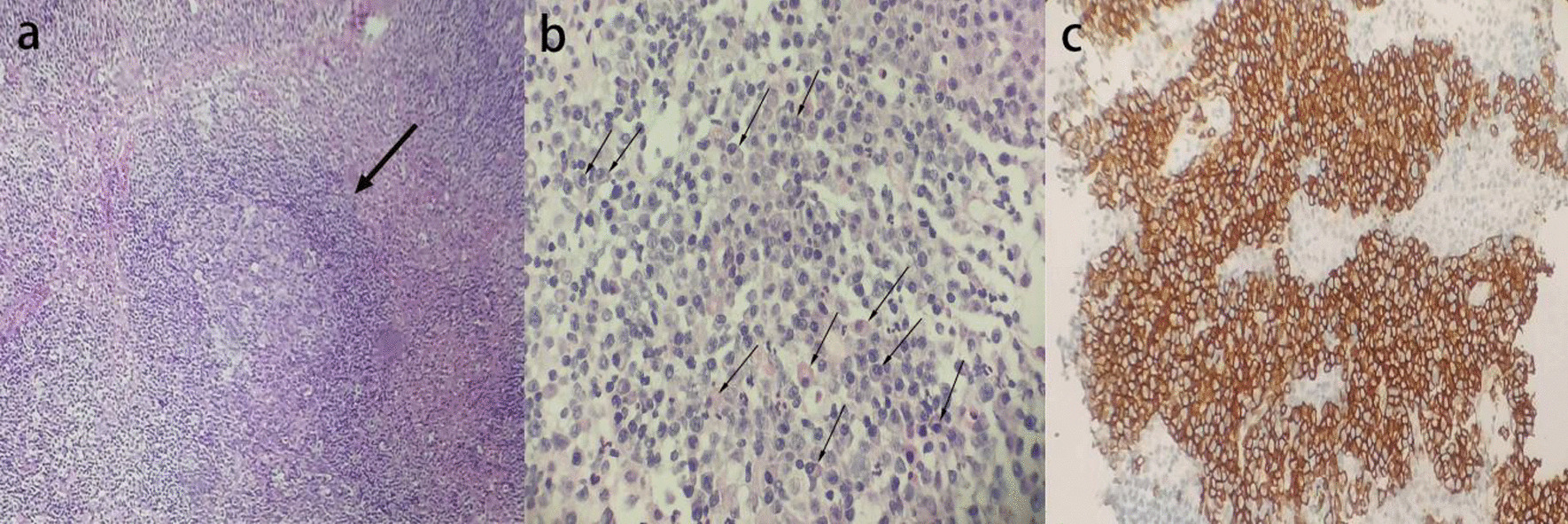
Lymph node biopsy showing features of Castleman disease of plasma cell type; **a** reactive hyperplasia of lymphoid tissue [arrow; hematoxylin and eosin (H&E) staining], **b** a large number of plasmacytic hyperplasia (arrows; H&E staining), **c** positive immunohistochemical staining for plasma cell markers: CD138

The patient was a 69-year-old male farmer and from Quzhou city, Zhejiang Province, China. He started experiencing persistent chest pain in March 2022, followed by intermittent of cough, expectoration, and fever. In September 2022, the patient visited the respiratory department because of worsening chest pain and cough accompanied by a low grade fever of 37.8 ℃. His medical history revealed that he had suffered from rheumatoid arthritis (RA) for 10 years and was prescribed tripterygium glycosides (20 mg three times per day) and prednisone (10 mg per day) to treat the disease since 2015. Tripterygium glycosides is a traditional Chinese herb that has the effects of antiinflammation and inhibition of cellular immunity. However, in August 2022, he stopped taking prednisone on his own because he was worried about its possible side effects. Physical examination revealed both lungs had coarse breath sounds, wet rales could be heard, and mild edema was observed in both lower limbs. In addition, there was a palpable lymph node without tenderness in the right axillary and other sites were not involved. The abdomen was flat without organomegaly. His initial blood tests revealed mild anemia, with low hemoglobin concentration (98 g/L, ref. range 130–175 g/L) and an elevated eosinophil ratio (7.2%, ref. range 0.5–5%). Other blood routine indicators, such as white blood cell (5.3 × 10^9^/L, ref. range 4–10 × 10^9^/L), neutrophil ratio (56.2%, ref. range 50–70%), lymphocyte ratio (29.1%, ref. range 20–40%), and blood platelet count (226 × 10^9^/L, ref. range 100–300 × 10^9^/L) were normal. C-reactive protein (58.2 mg/L, ref. range 0–5 mg/L), rheumatoid factor (437.2 IU/mL, ref. range 1–30 IU/mL), immunoglobulin G (IgG) (30.71 g/L, ref. range 8–18 g/L), and erythrocyte sedimentation rate (47 mm per hour, ref. range < 15 mm per hour) were elevated, while the total protein (56.5 g/L, ref. range 60–83 g/L), albumin (29.2 g/L, ref. range 35–53 g/L), and blood glucose (3.67 mmol/L, ref. range 3.9–6.1 mmol/L) were low. Serum levels of tumor markers, coagulation function, renal function, thyroid function, and IL-6 were all normal, and antibodies to the human immunodeficiency virus (anti-HIV) were negative. Computed tomography (CT) scan of the patient detected diffuse bronchial wall thickening and patchy area of air trapping consistent with small airway disease (Fig. 1a, b). Systemic lymph node color Doppler ultrasound showed enlargement of the right axillary lymph node (1.9 cm × 1.3 cm). Pulmonary function test showed mild small airway obstructive ventilation dysfunction (FEV_1_%: 75.5, MEF25%: 40.2, MEF50%: 35.3, MEF75%: 58.9) and moderate decrease in diffusion capacity. The patient was initially treated with antibiotics, but due to no improvement in symptoms, lymph node biopsy was performed to check for another differential diagnosis. The right axillary lymph node biopsy revealed reactive hyperplasia of lymphoid tissue with plasmacytic hyperplasia (Fig. 2a, b). Immunohistochemical staining showed clonal proliferation of plasma cells with the following findings: Ki-67 (germinal center, +), lambda (+), kappa (+), cyclin D1 (−), CD138 (plasma cell, +) (Fig. 2c), CD23 and CD21 [follicular dendritic cell (FDC), +], Bcl-2 (zone, +), Bcl-6 and CD10 (germinal center, +), CD79a and CD20 (B cell, +), and CD5 and CD3 (T cell, +).

Ultimately, the patient was diagnosed with the plasma cell type CD accompanied by BO. After successfully performing surgery to remove the enlarged lymph node in his right axilla, we suggested adjuvant therapy with glucocorticoid, but the patient declined because he was worried about its possible side effects. Hence the immunosuppressive medication tripterygium glycosides (20 mg three times per day) and leflunomide (20 mg per day) were administered postoperatively. He is currently asymptomatic and continues to take it. Bronchoscopy was performed at 6 months after surgery, and no abnormality was found. At present, he has been followed up for 11 months, pulmonary function test indicated mild small airway obstructive ventilation dysfunction (FEV_1_%: 75.9, MEF25%: 61.2, MEF50%: 31.6, MEF75%: 53.7) and normal diffusion capacity. The blood tests revealed the following: C-reactive protein (5.7 mg/L, ref. range 0–5 mg/L), erythrocyte sedimentation rate (8.6 mm per hour, ref. range < 15 mm per hour), IgG (15.62 g/L, ref. range 8–18 g/L), and IL-6 (0.97 pg/mL, ref. range 0–11.09 pg/mL). Rheumatoid factor was not tested. The patient is still under follow-up and enjoys a decent quality of life.

## Discussion

The etiopathogenesis of Castleman disease is poorly understood. UCD may be caused by clonal proliferation of tumor stromal cells and acquired gene mutations, and the most likely cell source is follicular dendritic cells [5, 6]. MCD originates due to immune dysfunction and excessive increase of cytokines caused by a variety of comprehensive factors, such as IL-6, human herpes virus-8 (HHV-8), and human immunodeficiency virus (HIV) [7]. About 70% of UCD pathological type is hyaline vascular, and diffuse proliferation of lymphoid follicles with enlarged tiny blood vessels can be observed under the microscope [8, 9]. In MCD, PC-CD and mixed CD are more common, and HV-CD accounts for about 20% of cases [10]. The clinical manifestations and pathological characteristics of CD are very similar to those of other immune diseases and neoplastic diseases, resulting in difficult diagnosis, underestimated incidence, and high misdiagnosis rate [11, 12].

Castleman disease can have a variety of complications, such as paraneoplastic pemphigus (PNP), bronchiolitis obliterans (BO), kidney injury, idiopathic thrombocytopenic purpura (ITP), and so on [13, 14]. Among them, BO and PNP are independent poor prognostic factors affecting the survival of patients [14, 15], with UCD being the most common.

Bronchiolitis obliterans (BO) is a chronic airflow obstructive pulmonary disease caused by bronchiolar inflammatory injury. The main clinical manifestations are chronic persistent cough, wheezing, and dyspnea [16]. According to relevant studies, the mortality of CD accompanied by BO is 48–85%, and the median overall survival time is 3 years [17]. In bronchial biopsy samples from these patients, IgG and complement have been seen to deposit linearly in the intercellular space and basement membrane area of bronchial epithelial cells, suggesting that the pathophysiology of BO may be associated with epithelial autoantibodies generated by CD tumor cells [18]. In addition, other immune damage mechanisms are involved in the pathogenesis of BO caused by CD. For example, CD8^+^ T-lymphocyte-mediated cytotoxicity may play an important role. Sano *et al*. [19] reported on the autopsy of patients diagnosed CD accompanied by BO that most of the inflammatory cells infiltrated in the bronchial wall of the lesion were CD8^+^ T lymphocytes. At present, there are few studies on the etiopathogenesis of BO caused by CD, which are limited to the biopsy and autopsy results of patients. It is not clear which cells or cytokines play a dominant role in the etiopathogenesis of BO, but we suspect that it is inseparable from the autoimmune disorders caused by CD.

The patient described here was admitted to the hospital with symptoms suggestive of pneumonia and had ineffective first treatment with antiinfective drugs. CD accompanied by BO was identified by a series of tests, and the tumor was surgically removed. Following surgery, the patient’s symptoms were partially resolved, and he is now fully capable of caring for himself. The patient was followed up about every 6 months after discharge to monitor laboratory examinations and pulmonary function. The prognosis of CD accompanied with BO is often poor, even after surgical resection of the tumor, and it is also difficult to prevent the progression of BO. In this case, the long-term oral tripterygium glycosides and prednisone before the diagnosis of CD did not inhibit the occurrence of BO, but may have delayed the progression. The patient was instructed to continue taking glucocorticoid after surgery, but he declined because he was worried about its possible side effects. Therefore, the immunosuppressive medication was administered postoperatively and followed up regularly after discharge. Now he is asymptomatic and still under follow-up.

CD is a rare disease, especially accompanied by BO. To the best of our knowledge, only six cases have been reported. Hassan *et al*. [20] reported two patients who received immunoglobulin, rituximab, fluticasone, azithromycin, and montelukast after surgery. After 11 months of follow-up, one of the patients had remission of symptoms. Wenhui *et al*. [21] described two patients who were treated with steroids, thalidomide, and immunosuppressive agents after surgery. One patient’s disease progressed after 2 years of follow-up, and the other patient’s disease progressed after 5 years of follow-up. Both ultimately had lung transplantation, and they gradually resumed their regular lives. Koichi *et al*. [22] reported a patient who received glucocorticoid administration after surgery and died of respiratory failure after 6 months of follow-up. Zhang *et al*. [23] also reported a patient who was treated with glucocorticoid after surgery, and his clinical symptoms were improved during the follow-up period. These cases indicate that for patients with CD with BO, postoperative adjuvant therapy is very important to try to prevent the progression of BO.

Patients with unicentric CD typically opt for complete surgical removal of the tumor, which has a 5-year survival rate of more than 90% and essentially has no impact on long-term survival [4, 8]. However, when the disease is associated with BO, it has a dismal prognosis and often needs to be treated with adjuvant therapy after surgery. If the patient’s condition worsens after surgery, lung transplantation, as well as adjuvant therapy, are better options. There are several reports that show that the patient’s condition improved after lung transplantation [21]. In this case, the patient received the immunosuppressive medication after refusing postoperative glucocorticoid therapy. He has been monitored for 11 months and is in good health. Continued close follow-up is required. MCD usually requires systemic treatment, but has a poor prognosis and a risk of transformation into lymphoma or plasmacytoma [24]. At present, rituximab chemotherapy is the first choice for the treatment of patients with MCD [12], but the long-term efficacy cannot be guaranteed due to the large toxicity [25].

## Conclusion

BO is the most significant poor prognostic factor and the leading cause of death in CD. Early detection of the disease, timely resection of the tumor, and effective adjuvant therapy may prevent the progression of BO. At present, most of the treatment methods for CD accompanied by BO are based on experience or previous case reports. Further research and accumulation of clinical case data are needed to explore more effective treatments. Clinicians should focus on educating patients regarding the course of the disease along with regular follow-ups to monitor clinical and laboratory changes. It is important to administer treatment promptly to enhance patients’ quality of life, their prognosis, and lower the death rate.

## Data Availability

All data and materials in this article are included in the manuscript.

## Abbreviations

CD: Castleman disease
BO: Bronchiolitis obliterans
CRP: C-reactive protein
IL-6: Interleukin-6
CT: Computed tomography
UCD: Unicentric Castleman disease
MCD: Multicentric Castleman disease
HV-CD: Hyaline vascular type Castleman disease
PC-CD: Plasma cell type Castleman disease
RA: Rheumatoid arthritis
Anti-HIV: Antibodies to the human immunodeficiency virus
HHV-8: Human herpes virus-8
HIV: Human immunodeficiency virus
PNP: Paraneoplastic pemphigus
ITP: Idiopathic thrombocytopenic purpura
